# Exposure to the Epstein–Barr Viral Antigen Latent Membrane Protein 1 Induces Myelin-Reactive Antibodies *In Vivo*

**DOI:** 10.3389/fimmu.2017.00777

**Published:** 2017-07-06

**Authors:** Yakov Lomakin, Georgii Pavlovich Arapidi, Alexander Chernov, Rustam Ziganshin, Evgenii Tcyganov, Irina Lyadova, Ivan Olegovich Butenko, Maria Osetrova, Natalia Ponomarenko, Georgy Telegin, Vadim Markovich Govorun, Alexander Gabibov, Alexey Belogurov

**Affiliations:** ^1^Shemyakin-Ovchinnikov Institute of Bioorganic Chemistry RAS, Moscow, Russia; ^2^Institute of Fundamental Medicine and Biology, Kazan (Volga) Federal University, Kazan, Russia; ^3^Moscow Institute of Physics and Technology, Dolgoprudny, Russia; ^4^Branch of Shemyakin-Ovchinnikov Institute of Bioorganic Chemistry RAS, Pushchino, Russia; ^5^Department of Immunology, Central Tuberculosis Research Institute RAS, Moscow, Russia; ^6^Research Institute of Physical Chemical Medicine, Federal Medical and Biological Agency, Moscow, Russia; ^7^Lomonosov Moscow State University, Moscow, Russia

**Keywords:** Epstein–Barr virus, latent membrane protein 1, multiple sclerosis, myelin basic protein, autoantibodies, cross-reactivity, epitope spreading

## Abstract

Multiple sclerosis (MS) is an autoimmune chronic inflammatory disease of the central nervous system (CNS). Cross-reactivity of neuronal proteins with exogenous antigens is considered one of the possible mechanisms of MS triggering. Previously, we showed that monoclonal myelin basic protein (MBP)-specific antibodies from MS patients cross-react with Epstein–Barr virus (EBV) latent membrane protein 1 (LMP1). In this study, we report that exposure of mice to LMP1 results in induction of myelin-reactive autoantibodies *in vivo*. We posit that chronic exposure or multiple acute exposures to viral antigen may redirect B cells from production of antiviral antibodies to antibodies, specific to myelin antigen. However, even in inbred animals, which are almost identical in terms of their genomes, such an effect is only observed in 20–50% of animals, indicating that this change occurs by chance, rather than systematically. Cross-immunoprecipitation analysis showed that only part of anti-MBP antibodies from LMP1-immunized mice might simultaneously bind LMP1. In contrast, the majority of anti-LMP1 antibodies from MBP-immunized mice bind MBP. *De novo* sequencing of anti-LMP1 and anti-MBP antibodies by mass spectrometry demonstrated enhanced clonal diversity in LMP1-immunized mice in comparison with MBP-immunized mice. We suggest that induction of MBP-reactive antibodies in LMP1-immunized mice may be caused by either Follicular dendritic cells (FDCs) or by T cells that are primed by myelin antigens directly in CNS. Our findings help to elucidate the still enigmatic link between EBV infection and MS development, suggesting that myelin-reactive antibodies raised as a response toward EBV protein LMP1 are not truly cross-reactive but are primarily caused by epitope spreading.

## Introduction

B cells and, particularly, antibodies are involved in pathogenesis of a variety of autoimmune disorders. These autoanibodies could be either a consequence of disease progression or directly participate in its development *via* different mechanisms such as antibody-mediated cell lysis, opsonization, cross-linking of Fc receptors, blocking or activation of cell receptors, and repair processes (1). Normally immature B cells recognizing self-antigens are forced to undergo receptor editing or eliminated during negative selection (2, 3). Therefore, the process of arising of such pathological self-reacting autoantibodies is still under investigation (4–8). In spite of the fact that polyreactive antibodies and B cell receptors (BCRs) may be considered as a common part of the normal immune system (9), they may arise as a result of different pathologies (10). One of the possible mechanisms to overcome the immune tolerance *post factum* is a cross-reactivity caused by a molecular mimicry. This hypothesis suggests that B cells initially recruited to neutralize external pathogens after somatic mutations and maturation start to produce low-to-middle affinity cross-reactive antibodies able to recognize self-antigens. Elimination of pathogens may conclusively shift humoral immunity toward further expansion of autoreactive B cells producing antibodies with increased affinity. Cross-reactivity of viral- and self-antigens is proposed as a probable triggering mechanism for many autoimmune disorders. Oxidative platelet fragmentation during immunologic thrombocytopenia is suggested to be caused by antibodies against GPIIIa cross-reactive with viral peptides hepatitis C and HIV-1 (11, 12). Kampylafka et al. reported that exposure to HTLV-1 virus can induce cross-reactive antibodies against aquaporin-4 triggering neuromyelitis optica (13). Lipo-oligosaccharides and gangliosides of *Campylobacter jejuni* may induce cross-reactive antibodies toward GM1, GM1b, and GAlNAc-GD1 in motor Guillain–Barre syndrome (14).

Multiple sclerosis (MS)—autoimmune neurodegenerative disorder with unknown etiology is associated with environmental factors, and especially with pathogen exposure. Numerous viral and bacterial infections are linked with MS pathology (15, 16). Molecular mimicry together with induction of systematic inflammation in the central nervous system (CNS) and destruction of the blood–brain barrier are proposed as the most likely inputs; however, distinct mechanisms of their involvement in disease progression are still under debate. Previously, we have shown that monoclonal human anti-myelin basic protein (MBP) immunoglobulins (IgGs) from MS patients are also reactive toward Epstein–Barr virus (EBV) latent membrane protein 1 (LMP1) (17). Recently, we reported that described molecular mimicry in MS patients is not unique but rather should be extended to a variety of viral and bacterial peptides (18, 19). The aim of the present study was to investigate (i) the possibility of developing myelin-reactive autoantibodies in response to viral antigen LMP1, (ii) whether such response is a characteristic of organisms predisposed to autoimmune abnormalities, and (iii) how the manner of contact with an antigen can affect development of cross-reactive antibodies. In this study, we present evidence that a significant part of the antibodies initially developed *in vivo* against viral pathogens can recognize the neuronal autoantigen MBP.

## Animals and Methods

### Antigens for Immunization

Bovine MBP was purified as described previously (20); bacterially expressed recombinant LMP1 N- and C-domains (19) or CTAR1/2/3 fragments were purified using Ni-NTA immobilized metal affinity capture and further subjected to size-exclusion chromatography on a Superdex 75 column (GE Healthcare Life Sciences); ovalbumin (OVA) was purchased from Sigma-Aldrich (A5503). All antigens were tested for endotoxin presence with a commercially available endotoxin detection assay (Lonza, Switzerland).

### Animals

BALB/c and SJL mice were from Animal Breeding Facility, Branch of Shemyakin-Ovchinnikov Institute of Bioorganic Chemistry RAS (Pushchino, Russia), accredited AAALAC International (file number 001093). All studies involving experimental animals were approved by the Institutional Animal Care and Use Committees of Pushchino Branch of Shemyakin-Ovchinnikov Institute of Bioorganic Chemistry (Pushchino, Russia).

### Antigen Exposure

#### Chronic Immune Response (CIR) Model

Female SJL mice (6–8 weeks old) were immunized subcutaneously with 100 µl of emulsion containing 50 µl complete Freund adjuvant (Sigma-Aldrich) with 1 mg/ml *Mycobacterium tuberculosis* and 50 µg of MBP, LMP1, or OVA in PBS. One hundred microliters of emulsion was distributed over two sites (i.e., ~0.05 ml per site): one along the midline of the back between the shoulders, and other on the lower back. Mice were also injected with 200 and 100 ng pertussis toxin i.v. on the day of, and 2 days following the first immunization, respectively. The second immunization was performed in the same fashion 7 days after the first immunization. Diminished concentration of *M. tuberculosis* (1 mg/ml instead of 4 mg/ml) and pertussis toxin in comparison with the classical protocol of experimental autoimmune encephalomyelitis (EAE) induction (21) was used with premeditation to avoid EAE development and bystander T-cell activation.

#### Multiple Acute Subsequent Immunization (MASI) Model

**Figure 1:**
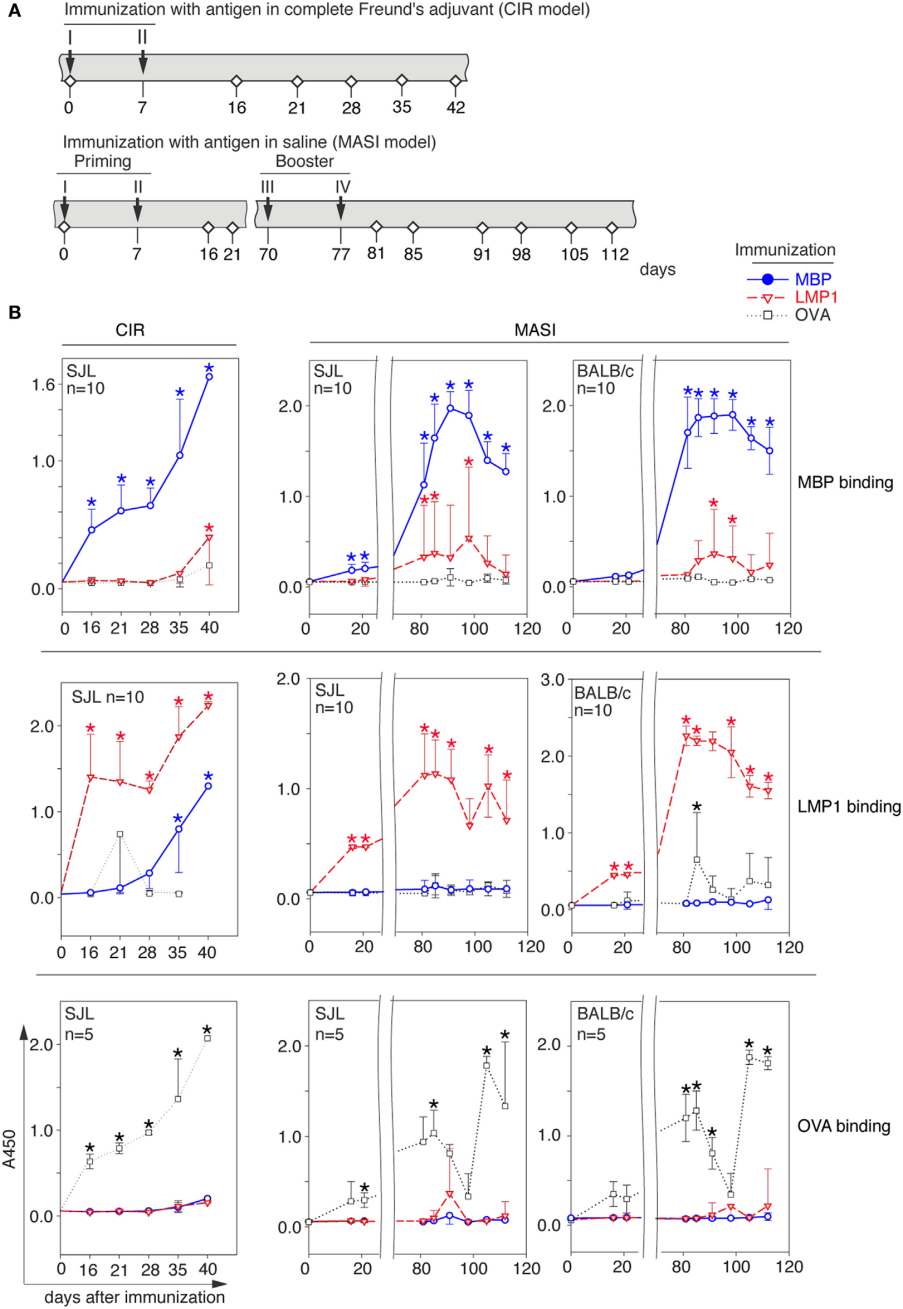
Myelin-reactive antibodies are induced *in vivo* upon immunization with viral antigen. **(A)** Immunization schemes representing chronic immune response (CIR) and multiple acute subsequent immunization (MASI) models. Timeline indicates immunizations (designated by roman numbers and arrows) and blood sample collection from vena ophthalmica (designated by white rhombs). **(B)** Analysis of presence of anti-myelin basic protein (MBP) (top), anti-LMP (middle), and anti-ovalbumin (OVA) (bottom) antibodies in serum of BALB/c and SJL mice immunized with MBP (blue line), latent membrane protein 1 (LMP1) (red line), or OVA (black dashed line) according to CIR (left) or MASI (right) model. Data represent average and SD. Asterisk denotes statistically significant difference of anti-MBP and anti-LMP1 antibody titer in serum of MBP- or LMP1-immunized mice in comparison with a control (OVA-immunized mice) or anti-OVA antibody titer in serum of OVA-immunized mice in comparison with a control with a MBP- and LMP1-immunized mice.

Female SJL or BALB/c mice (6–8 weeks old) were immunized subcutaneously with 100 µl of saline containing 50 µg of MBP, LMP1, or OVA in PBS. One hundred microliters of this solution was distributed over two sites (i.e., ~0.05 ml per site): one along the midline of the back between the shoulders and another on the lower back. Mice were also injected with 200 and 100 ng of pertussis toxin i.v. on the day of and 2 days following the first immunization, respectively. Second immunization was performed in the same fashion on the day 7 after the first immunization. Booster immunization was performed in a similar manner on days 70 and 77 following the first immunization. Serum was taken each 5–8 days (per the scheme in Figure 1A), and stored at −20°C until cross-reactivity testing. All mice were euthanized at the end of experiments.

### T Cell Proliferation Assay

Mice were sacrificed 35 days postimmunization. Mononuclear cells (MNC) from lymph nodes and spleen were passed through a 40-mm nylon mesh, enriched with a Ficoll gradient centrifugation and adjusted to a concentration of 2 × 10^6^ cells/ml. Antigen-induced T-cell proliferation was measured per the following procedures. MNC were stained with CFDA-SE [5 µM, 8 min, room temperature (RT)] and cultured in 96-well plates (2 × 10^5^ cells/well). Antigens (MBP, LMP1, OVA) were added to the culture at 1:1 ratio to the final concentration of 15 µM. Three days later cells were harvested and stained with APC-anti-CD8 and PerCP-Cy5.5-anti-CD4 mAbs (Biolegend, San Diego, CA, USA). Proliferation of CD4^+^ and CD8^+^ cells were assessed by flow cytometry based on CFSE dilution. All experiments were performed on BD FACS Canto II (BD Biosciences, San Jose, CA, USA). Unstimulated cells and cells stimulated with immobilized anti-CD3 mAb were used as negative and positive controls, respectively.

### Determination of Antibody Cross-reactivity

MaxiSorp 96-well plates (Nunc, Denmark, Roskilde) were coated with a test antigen solution (LMP1, MBP, OVA, recombinant LMP1 fragments, or recombinant MBP fragments; 5 µg/ml in 50 µl/well) in 100 mM carbonate buffer, pH 9.0 at 4°C overnight, and washed with three portions of a wash buffer (PBS with 0.1% Tween 20, pH 7.4, 300 µl/well). Unless otherwise specified, this washing was performed after each step. The wells were then blocked with 250 µl of dry milk in carbonate buffer and incubated at 37°C for 1 h. Serum samples were diluted in PBS containing 0.5% dry milk and 0.05% Tween 20. Respective monoclonal or polyclonal antibodies were used for each antigen as a positive control. The plates were incubated with primary antibodies at 37°C for 1 h. Then, 50 µl of HRP-conjugated anti-mouse anti-Fab antibodies (1:5,000) was added to each well, and the plates were incubated at 37°C for 1 h. After washing with five portions of the wash buffer, 50 µl of tetramethyl benzidine (Amresco, Solon, OH, USA) was added to each well, and the plates were placed in the dark for 5–15 min. The reaction was stopped by adding 10% phosphoric acid (50 µl/well). The OD_450_ values were measured on a Varioskan Flash microplate reader (Thermo Scientific, Waltham, MA, USA).

### IgG Enrichment on MBP and LMP1 Sepharose

Myelin basic protein or LMP1 was immobilized on NHS-activated Sepharose HP (GE Healthcare, USA) per the manufacturer’s instructions. Briefly, after washing steps with 1 mM HCl, antigen in buffer containing 0.2 M NaHCO_3_, 0.5 M NaCl (pH 8.3) was added to resin at ratio 1:1 and incubated for 4 h at RT. To block non-reacted groups on resin, it was kept in 0.5 M ethanolamine for 2 h. Final washes were performed serially in 0.1 M Tris–HCl (pH 8.5) and 0.1 M acetate buffer supplemented with 0.5 M NaCl, pH 4.5. Purified IgGs from MBP or LMP1-immunized mice were precipitated with Sepharose-immobilized MBP or LMP1 per the standard immunoprecipitation protocol (initially we purified IgGs from combined serum of MBP- or LMP1-immunized mice with cross-reactive responses against MBP and LMP1). Briefly, IgGs in PBS (300 µl at concentration of 0.5 mg/ml) were added to Sepharose beads (120 µl of dry Sepharose), adjusted to the final volume of 0.5 ml and incubated at +10°C during 2 h with constant gentle shaking. Flow through was collected for further analysis. Non-specifically bound IgGs were removed by triple washing with PBS. Elution of MBP-specific or LMP1-specific IgGs was performed with 0.1 M Gly–HCl, pH 2.5 and subsequent neutralization with 1.0 M Tris–HCl, pH 9.0. For MS/MS analysis, antibodies were eluted with sample buffer and subjected to the polyacrylamide gel electrophoresis, respective bands (approximately 50 kDa—for heavy chain) were cut from the gel, alkylated with iodoacetamide, digested by trypsin and subjected to further analysis.

### Mass-spectrometry Analysis

Analysis was performed on a TripleTOF 5600+ mass-spectrometer (Sciex, Canada) coupled with a NanoLC Ultra 2D+ nano-HPLC system (Eksigent, USA). The HPLC system was configured in a trap-elute mode. Samples were eluted through a 3C18-CL-120 separation column (3 µm, 120 Å, 75 μm × 150 mm; Eksigent, USA) at a flow rate of 300 nl/min during 120 min. Information-dependent mass-spectrometer experiment included one survey MS1 scan followed by 50-dependent MS2 scans. Detailed methodology is included in the Supplemental Information.

### Peptide Identification

Raw MS/MS analyses were converted to mgf peak lists with the ProteinPilot (version 4.5, Sciex, Canada). For this procedure, ProteinPilot was run in identification mode with the following parameters: trypsin digestion, cys alkylation by iodoacetamide, TripleTOF 5600 instrument, thorough ID search with detected protein threshold 10.0% against UniProtKB data base (22), taxon *Mus musculus*. To identify peptides corresponding to the murine IgG CDRs, resulted mgf peak lists were analyzed by PEAKS Studio 7.5 (Bioinformatics Solutions Inc., Canada) with the following parameters: data refinement: min charge: 1, max charge: 4; *de novo*: precursor mass: 10.0 ppm, fragment ion 0.01 Da, no enzyme or PTM; general option: report up to 30 candidates per spectrum. Filtration of results was accomplished utilizing ALC (average local confidence) values calculated by PEAKS Studio, which corresponds to the average confidence level of the determined amino acid. The following criteria were used: interpretation of spectra with ALC >80%, all interpretations with ALC <50% were withdrawn. Thus, 5,783 spectra were interpreted. The amount of unique peptide sequences were estimated as 87,678, every spectrum was interpreted with up to 30 different sequences. If at least one interpretation corresponded to the known murine protein [UniProtKB (22)] or to the constant fragment of the murine Ig [IMGT (23)], the spectrum with all linked identifications was withdrawn from the list. Next, filtered peptides were tested if they contain sequence tags corresponding to the three to four amino acids of framework Ig regions or N-terminus of Ig D-segment. Identified sequences should additionally contain lysine or arginine on the C-terminus, internal lysines and arginines were not taken into consideration. Spectra still having more than three interpretations at this stage were withdrawn. More than 90% of obtained spectrums had only one possible identification.

Identified CDR sequences corresponding to the MBP-/LMP1-binding or cross-reactive antibodies were aligned and clustered utilizing Unipro UGENE v1.13.0 (24). A consensus motif was created for each cluster using WebLogo (25) software.

### Experimental Validation of the *De Novo* Identified Peptides

To verify 11 CDR fragments identified *de novo*, predicted peptides were synthesized (Shanghai Ruifu Chemical Co., Ltd.), and their MS2 spectra were recorded. Spectral libraries were created based on mass-spectrometrical analysis of the synthetic peptides by SpectraST (Institute for Systems Biology, USA). Identifications of spectra from experimental samples were repeated against these spectral libraries by SpectraST. In addition, six of these peptides were synthesized with C-terminal Lys-biotin for binding analysis with MBP and LMP1 utilizing ELISA and WB techniques.

### Statistical Analysis

Data were analyzed using the Sigma-Plot 12.5 software. All samples were measured in triplicate (technical replicates). The amount of tested animals (biological replicates) is designated on each figure describing the experiment (*n* = x).

## Results

### Immunization of BALB/c and SJL Mice with LMP1 Results in Induction of Myelin-Reactive Autoantibodies

In our study, we used two immunization schemes (Figure 1A): (i) immunization with antigen in complete Freund’s adjuvant for induction of CIR and (ii) immunization in physiological buffer where “priming” and “booster” stages each included two injections separated by 2 months (MASI). SJL and BALB/c mice were immunized with LMP1, MBP, or irrelevant protein OVA. At indicated time points, serum antibodies from immunized mice were tested for antigen binding (Figure 1B). Antibodies from LMP1-immunized mice bound both, initial antigen and MBP (though with less affinity), regardless the immunization scheme, whereas in MBP-immunized mice antibodies toward LMP1 arose only in the CIR model mice. Levels of anti-MBP antibodies in mice immunized with LMP1 per the priming-booster scheme were similar in SJL and BALB/c mice. Remarkably, neither LMP1- nor MBP-immunized mice showed induction of reproducible levels of anti-OVA antibodies. In line with this observation, no reasonable level of antibodies against either LMP1 or MBP was detected in OVA-immunized mice (Figure S1A in Supplementary Material). To compare immunogenic epitopes of LMP1 in LMP1- and MBP-immunized mice, we checked antibody levels in these mice toward C- and N-terminal parts of LMP1 and its C-terminal fragments (CTAR elements) (Figure S1B in Supplementary Material). The C-terminal region of LMP1 was immunodominant regardless of which antigen was used for immunization. Nonetheless, the level of antibodies against CTARs was considerably higher in LMP1-immunized mice compared to MBP-immunized mice (Figure S1C in Supplementary Material).

### Myelin-Specific Autoantibodies Induced by LMP1 Immunization Are Caused by Epitope Spreading and Are Only Partially Cross-reactive

**Figure 2:**
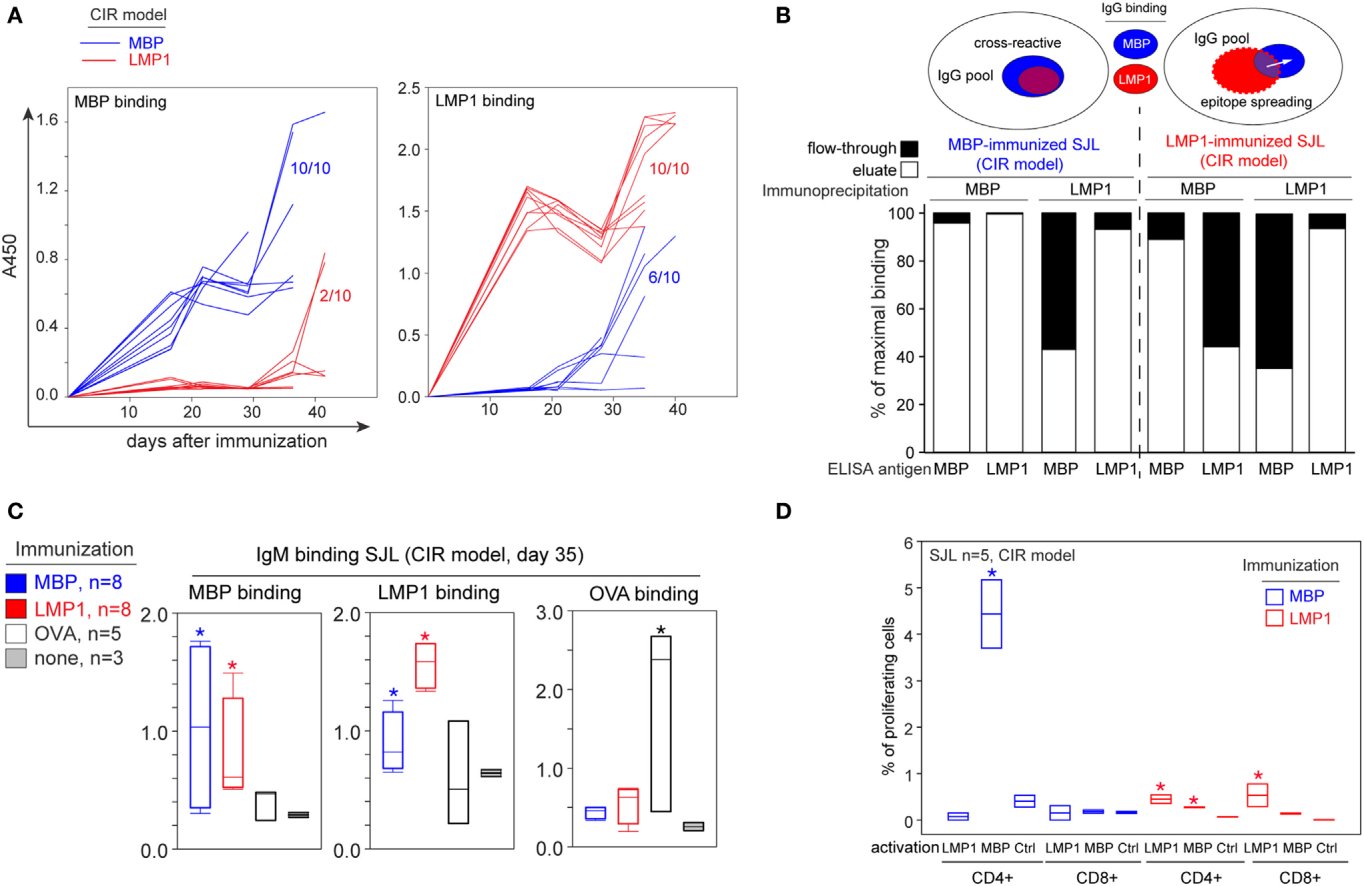
Myelin-reactive antibodies observed in latent membrane protein 1 (LMP1)-immunized mice are not solely cross-reactive but partially caused by epitope spreading. **(A)** Individual profiling of induction of anti-myelin basic protein (MBP) (left panel) and anti-LMP1 (right panel) serum antibodies in SJL mice immunized with MBP (blue line) or LMP1 (red line) according to the chronic immune response (CIR) model. The numbers indicate the amount of mice with positive IgG response toward antigen under investigation. **(B)** Purified IgGs from MBP-immunized (left) or LMP1-immunized (right) SJL mice were enriched on MBP or LMP1 as indicated in graph “Immunoprecipitation” and further fractions were analyzed for binding of MBP and LMP1. Bars represent binding of eluate (white bars) and flow through (black bars) in comparison with binding of unfractionated IgGs for each sample. Upper scheme demonstrates overlapping of anti-MBP and anti-LMP1 IgG repertoires in MBP- and LMP1-immunized SJL mice according to the results of immunoprecipitation. **(C)** Analysis of presence of anti-MBP (left panel), anti-LMP1 (middle panel), and anti-ovalbumin (OVA) (right panel) IgM in SJL mice immunized with MBP (blue bars), LMP1 (red bars), and OVA (white bars), or non-immunized SJL mice (gray bars). Blood samples were collected on day 35 after immunization. Asterisk denotes statistically significant difference with a control (non-immunized mice). **(D)** Proliferative response of CD4/CD8-positive T cells derived from SJL mice immunized with MBP (blue bars) or LMP1 (red bars) and stimulated *in vitro* with the indicated antigens. Shown are percentages of proliferating cells determined in CFSE dilution assay. Ctrl corresponds to negative control (cell culture medium). Bars represent interquartile range, SD is indicated. Asterisk denotes statistically significant difference with a negative control.

In the CIR model, 6/10 MBP-immunized mice developed antibodies toward LMP1 and antibodies from only 2/10 LMP1-immunized mice bound MBP (Figure 2A). We therefore posited if the discovered bystander activity toward LMP1 or MBP increases mainly due to the production of autoantibodies’ recognizing both antigens simultaneously or if it is a consequence of epitope spreading. To elucidate this point, we performed enrichment of antibodies on beads-immobilized MBP or LMP1 followed by ELISA. Thus, we showed that in MBP-immunized SJL mice, almost all antibodies toward LMP1 were in the fraction of anti-MBP antibodies, while for LMP1-immunized SJL mice more than half of MBP-binding antibodies did not bind LMP1 (Figure 2B). We next measured levels of IgM antibodies and T cell response in immunized and intact mice. Remarkably, similar to IgG, the level of IgM against LMP1 and MBP was readily detected in SJL mice immunized with MBP and LMP1, respectively (Figure 2C). The specificity of T cell response mounted following the immunization with MBP or LMP1 was assessed by analyzing the proliferation of spleen and lymph node cells derived from immunized SJL mice. Isolated cells were restimulated *in vitro* with MBP or LMP1 antigens and the percentage of responding cells was assessed by CFSE dilution assay. Significant proliferation was observed for CD4^+^ T cells derived from MBP-immunized mice and restimulated with MBP antigen. Activation of T cells from LMP1-immunized mice was less pronounced, but still detectable. The CD4^+^ cells from LMP1-immunized mice proliferate in response to LMP1 and MBP, and CD8^+^ cells were activated after incubation with LMP1 (Figure 2D).

### *De Novo* LC-MS/MS Sequencing of Myelin- and LMP1-Reactive Antibodies Revealed Structural Peculiarities of Their CDR3, Depending on Immunization Agent

**Figure 3:**
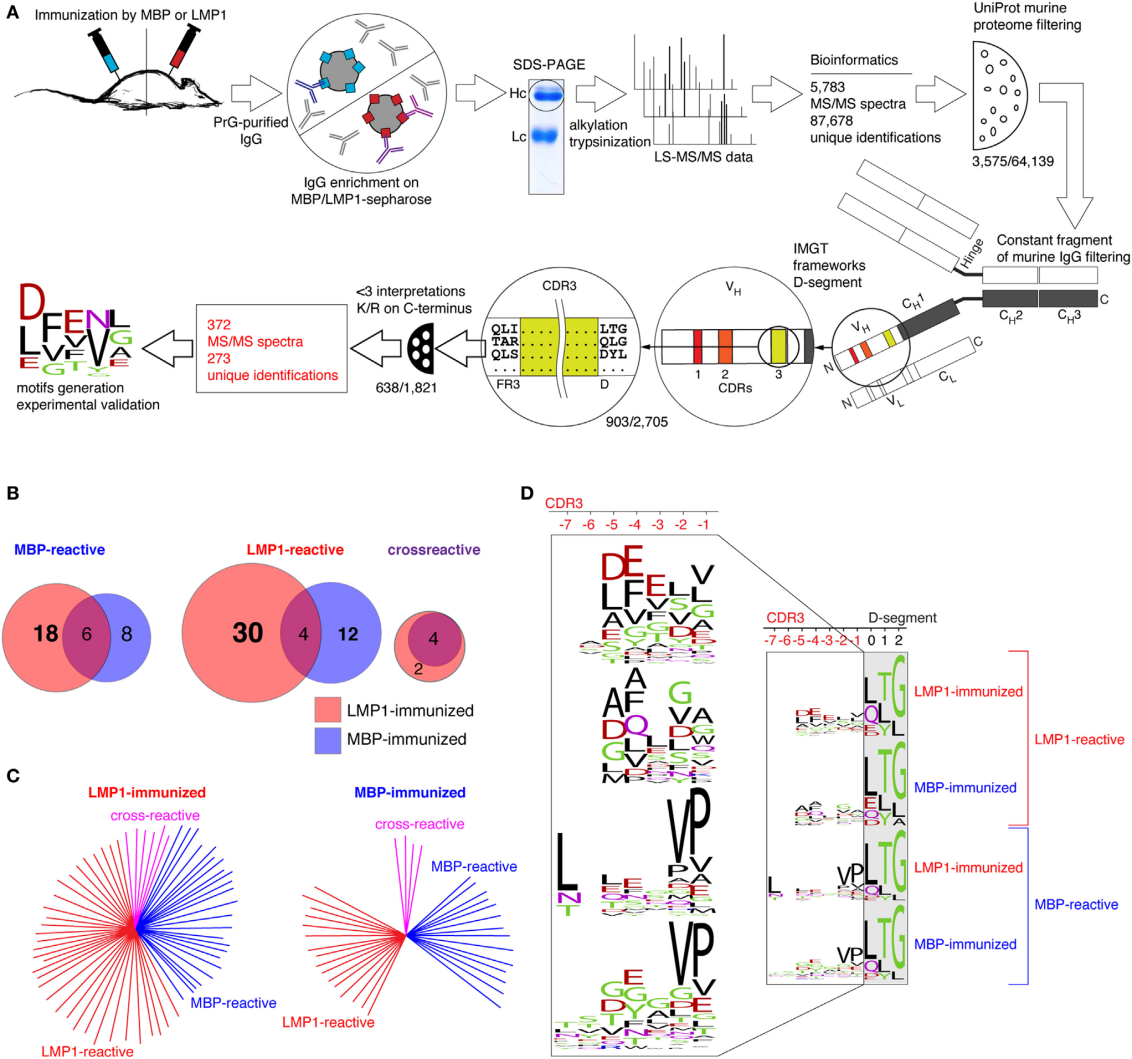
Comparative analysis of repertoires of CDR3 sequences corresponded to anti-myelin basic protein (MBP), anti-latent membrane protein 1 (LMP1), and cross-reactive antibodies. **(A)** Workflow of the *de novo* sequencing and data analysis algorithm (for detailed description please refer to the Sections “Results” and “Animals and Methods”). **(B)** Amount of unique CDR3 sequences corresponded to MBP-, LMP1- or cross-reactive IgG from MBP-immunized (blue circles) and LMP1-immunized (red circles) mice. **(C)** Amount of unique CDR3 sequences corresponded to MBP-reactive (blue), LMP1-reactive (red), or cross-reactive (pink) IgG from MBP-immunized and LMP1-immunized mice. **(D)** Amino acid sequences of identified super motifs of CDR3 fragments corresponded to LMP1- and MBP-reactive antibodies from LMP1- and MBP-immunized mice.

To characterize the repertoire of viral- and myelin-reactive antibodies, we performed LC-MS/MS analysis of four separate tryptic hydrolyzates of purified IgGs from MBP- and LMP1-immunized mice enriched on MBP or LMP1 proteins immobilized on carboxylated microspheres. Existing databases covering sequences of mouse immunoglobulins are evidently insufficient, therefore we performed *de novo* MS deconvolution of variable IgG fragments (Figure 3A). Initially, we collected 5,783 MS/MS spectra corresponding to the 87,678 unique peptide sequences, as on average, for each spectrum, up to 30 different amino acid sequences could be interpreted. Obtained data were filtered against peptides from known murine proteins and constant fragments of the murine Ig. Finally, resting identifications were checked if they contained sequence tags corresponding to the three to four amino acids of framework Ig regions or N-terminal parts of Ig D-segments. Thus, a number of unique sequences were reduced to 270 peptides corresponding to the 347 MS/MS spectra. Identified N- and C-terminal fragments of CDR3 of MBP- and LMP1-binding- or cross-reactive antibodies are listed in Figure S2 in Supplementary Material and Table 1. It is evident that a variety of unique CDR3 sequences, corresponding to both LMP1- and MBP-binding antibodies, in LMP1-immunized mice are considerably higher than that seen after MBP-immunization (Figures 3B,C). Generated super motifs of CDR3 of MBP- and LMP1-reactive antibodies revealed negatively charged clusters in [-3]-[-5] positions in LMP1-reactive antibodies from LMP1-immunized mice, and hydrophobic clusters in C-terminal positions of MBP-reactive antibodies from either MBP- or LMP1-immunized mice (Figure 3D). To verify this observation, we synthesized 11 peptides corresponding to six spectra representing possible CDR sequences. According to MS2 spectra of synthesized peptides (Figure S3 in Supplementary Material), only one *de novo* predicted CDR3 sequence was misinterpreted. To elucidate whether identified peptides corresponding to the H-CDR3 sequences recognize MBP or LMP1, we performed immunoprecipitation followed by ELISA. We revealed that one fragment (TYGGTFTDYLYNWVK), corresponding to the H-CDR3 of the MBP-binding antibody from LMP1-immunized mouse, recognized MBP in both utilized assays (Figure S4 in Supplementary Material).

**Table 1:** CDR3 sequences discovered by *de novo* MS–MS sequencing.

Origin (immunization antigen)	Predicted specificity	CDR3 sequence	MS–MS verification	Synthesized biotinylated peptides^a^	Experimentally confirmed binding		ID
					Myelin basic protein (MBP)	Latent membrane protein 1 (LMP1)	
LMP1	MBP	GASDGSTDYGLL	Passed	GASDGSTDYGLLQLGGSRK (Bio)	–	–	P1
LMP1		APSTYGGGTT			n/d	n/d	
LMP1		FLEQENAGV			n/d	n/d	
LMP1		LATSVYAST	Passed	LATSVYASTLTGK (Bio)	+/−	–	P2
MBP/LMP1		FLEQENQV			n/d	n/d	
MBP/LMP1		DLSTNTAM			n/d	n/d	
MBP/LMP1		VNVDEVGE			n/d	n/d	
LMP1		TYGGTFTY			n/d	n/d	
LMP1		TYGGTFT	Passed	TYGGTFTDYLYNWVK (Bio)	+	–	P3

LMP1	LMP1	FNQGLSER			n/d	n/d	
LMP1		YDEAPSGGVA			n/d	n/d	
LMP1		ARALETVT	Failed		n/d	n/d	
LMP1		LNALETVT			n/d	n/d	

MBP/LMP1	Cross-reactive	NTDGSTDYGLL	Passed	NTDGSTDYGLLQLK (Bio)	–	–	P4
MBP/LMP1		GDGSGTSFL	Passed	GDGSGTSFLLTGK (Bio)	–	+/−	P5

*^a^Fragment of D-segment from identified peptide sequence is underlined*.

*n/d, no data*.

## Discussion

In the present study, we used two different schemes of immunization to determine if the duration and frequency of contact of immune system with antigen could influence the affinity, polyreactivity, and cross-reactivity of developing antibodies. Immunization in complete Freund’s adjuvant, which enables preserving the antigen in mice up to several weeks, was used for induction of CIR, imitating exposure to the viral antigen during infectious mononucleosis—severe clinical manifestation of EBV. MASI mode was used to imitate several consequent EBV infections. The aim of our study was to examine actual B cell response toward viral antigens; therefore, we use reduced dosage of pertussis toxin and *M. tuberculosis* during immunization procedures to prevent invasion of the immune system into the CNS and subsequent development of EAE. Indeed, mice in all groups showed no relevant clinical scores (data not shown); at the same time, we suggest that endogenous myelin antigens were at least in part accessible for immune cells during B cells maturation. Our data generally show that detected antibody response was similar in SJL and BALB/c mice. Therefore, induction of antibodies toward MBP or LMP1 on the background of immunization with LMP1 or MBP, respectively, is not prerogative of autoimmune-predisposed organisms but is rather only a general property of the immune system.

**Figure 4:**
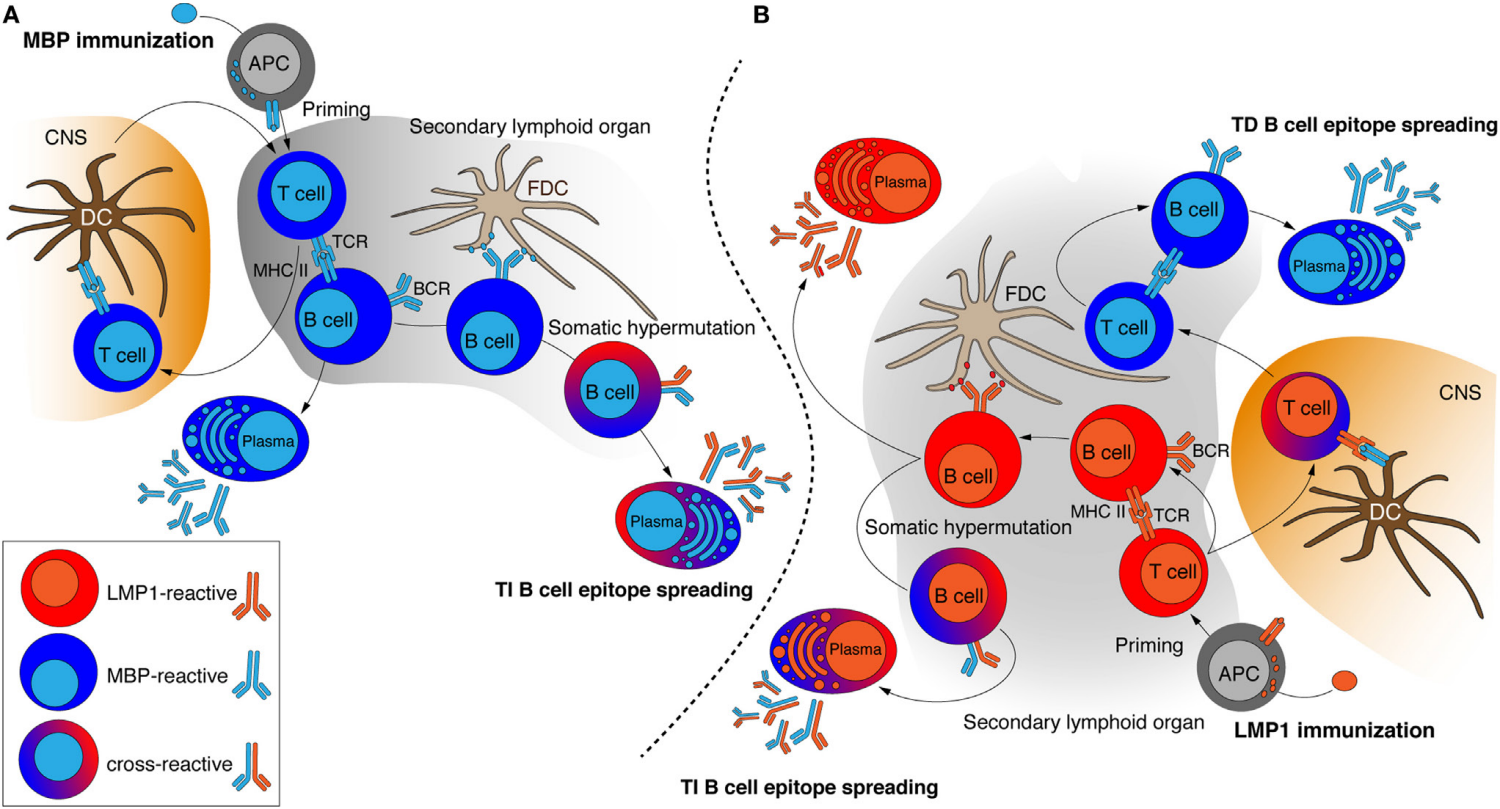
Probable mechanism of induction of cross-reactive antibody response in the myelin basic protein (MBP)-immunized **(A)** and latent membrane protein 1 (LMP1)-immunized **(B)** mice. Antigen-activated T cells establish stable interactions with B cells and therefore enable B cells to receive helper signals from cognate CD4^+^ T cells and further undergo proliferation that finally resulted in appearance of antibody-producing plasma cells. Induction of cross-reactive antibodies may be caused by BCR diversification through somatic hypermutation accompanied by FDCs or activation by myelin-specific T cells that are primed directly in CNS. APC, antigen-presenting cell; DC, dendritic cells; FDC, follicular dendritic cell; TCR, T cell receptor; BCR, B cell receptor; MHC II, major histocompatibility complex class II molecules; TI, T cell-independent; TD, T cell-dependent; CNS, central nervous system.

Chronic immune response mode in comparison with MASI resulted in more rapid but concurrently more occasional appearance of MBP-reactive antibodies in LMP1-immunized mice. These data support previous observations that infectious mononucleosis, which is characterized by massive and prolonged exposure to EBV, increases risk of MS development (26, 27). Because EAE in SJL mice is shown to be driven by Th1 T cells, we also tested whether T cell response is present. We observed distinct MBP-driven CD4^+^ T cells activation in MBP-immunized mice, whereas diminished activation of CD4^+^ T cells by both, LMP1 and MBP, was detected in LMP1-immunized mice. We therefore suggest that induction of LMP1-reactive antibodies in MBP-immunized mice is seemed to be T cell-independent and probably is caused by Follicular dendritic cells (FDCs) (28) inducing somatic hepermutations in B cells leading to appearance of anti-MBP antibodies with occasional LMP1-binding capacity (Figure 4A). In contrast, in LMP1-immunized mice induction of MBP-reactive antibodies may proceed not only through FDC-dependent mechanism but also involve activation by T cells that are primed by myelin antigens directly in CNS (29) (Figure 4B).

Generally, induction of antibodies toward “cross-antigens” was reduced and significantly delayed compared to development of antibodies against injected antigen. In MBP-immunized SJL mice, relative affinity of autoantibodies to LMP1 30 days after initial antigen exposure reached affinity of antibodies toward the parental antigen, whereas even after 40 days in LMP1-immunized mice, anti-MBP antibodies were still less mature in comparison to LMP1-reactive antibodies (Figure S5 in Supplementary Material). Interestingly, in MBP-immunized mice, antibodies to LMP1 were raised in the CIR but not in MASI model. To explain this observation, we suggest that in CIR model the immunization probably leads to the more explosive, massive, and uncontrolled response, which results in developing of antibodies with more promiscuous specificity. The amount of identified CDRs corresponded to LMP1- and myelin-reactive antibodies was approximately doubled in LMP1-immunized mice compared to MBP-immunized mice. Therefore, it is possible to speculate that exposure of mice to EBV antigen can provide a more variable and representative, but less mature repertoire of immunoglobulins.

The epitope profiling of anti-LMP1 antibodies in MBP- and LMP1-immunized mice revealed that their maturation vectors differ. In this study, we present evidence that anti-LMP1 antibodies in MBP-immunized mice are rather anti-MBP antibodies that obtain ability to cross-react with LMP1 during maturation process. This observation is in line with data suggesting that natural IgM in non-immunized animals able to recognize some conservative pathogen antigens (30–32). In contrast, induction of anti-MBP antibodies in LMP1-immunized mice is evidently a rare process, which requires epitope spreading. One may speculate that negatively charged clusters of aspartate and glutamate in the CDR of LMP1-reactive antibodies may allow these antibodies to bind MBP with low affinity, with subsequent appearance of intermediate cross-reactive antibodies that in turn mature to MBP-reactive antibodies with hydrophobic C-terminal clusters. According to our data, it seems that CDR3 sequences found in LMP1- or MBP-specific antibodies are encoded by somatically mutated genes. However, because of the combinatorial and non-templated nature of the mechanisms that generate the CDR-H3, it is the most diverse component in terms of length and sequence of the antibody H-chain repertoire (33, 34). Therefore, in our case because of incomplete sequence of CDR3 and lack of comprehensive data related to variable part of antibody heavy chain, it is inconveniently to accurately attribute identified CDR3 sequences to a certain germline. Probably, future experiments that will combine LS-MS/MS analysis of the antigen-specific antibodies with NGS of B-memory cells would be able to solve this problem (35).

It should be emphasized that in the case of MBP-immunized mice, *de novo* sequencing is not fully correlated with ELISA. Mass-spectrometry studies revealed only a few common CDRs between anti-LMP1 and myelin-reactive antibodies (Figure S2 in Supplementary Material), whereas ELISA demonstrated truly cross-reactive antibodies capable of binding MBP and LMP1 simultaneously. This could be explained by the difficulties of quantitative IgG profiling by mass spectrometry, while ELISA reflect ratio of MBP- and LMP1-reactive antibodies in regard with their affinity toward the antigen. We therefore suggest that despite existence of antibodies binding exclusively LMP1 in MBP-immunized mice, their affinity or effective concentration in serum is vanishingly small.

Our data uncover some peculiarities of EBV infection in MS development. First, chronic contact with viral antigen induced a switch of B cells to myelin antigen more efficiently than multiple rapid exposures to EBV. Second, even in inbred animals, which are genetically almost identical, such switching was observed only in 20–50% of animals, indicating that this occurred by randomly rather than systematically. These findings are interesting considering the existing dilemma observing that most the population, that is evidently significantly more heterogeneous than inbred animals, is infected with EBV, but only 1 person in 1,000–10,000 will develop MS. Further studies should resolve if B cells producing anti-LMP1 antibodies during MS should be considered as unspecific traces of massive reactions of the immune system toward myelin antigens, or are primary seeds that years prior shifted the balance toward autoimmunity. In turn, high-throughput sequencing of CDRs repertoire to identify B cells with potential to transform into self-reactive lymphocytes may serve as an additional prognostic MS criterion.

## Ethics Statement

BALB/c and SJL mice were from Animal Breeding Facility, Branch of Shemyakin-Ovchinnikov Institute of Bioorganic Chemistry RAS (Pushchino, Russia), accredited AAALAC International (file number 001093). All studies involving experimental animals were approved by the Institutional Animal Care and Use Committees of Pushchino Branch of Shemyakin-Ovchinnikov Institute of Bioorganic Chemistry (Pushchino, Russia).

## Author Contributions

YL, GA, RZ, NP, VG, AG, and AB designed research; YL, GA, AC, RZ, ET, and IL performed research; YL, GA, IB, MO, GT, VG, AG, and AB analyzed data; and YL, AG, and AB wrote the paper.

## Conflict of Interest Statement

The authors declare that the research was conducted in the absence of any commercial or financial relationships that could be construed as a potential conflict of interest.
