# Supplementary material for: Exposure to the Epstein–Barr Viral Antigen Latent Membrane Protein 1 Induces Myelin-Reactive Antibodies In Vivo
Source: Front Immunol. 2017 Jul 6;8:777. doi: 10.3389/fimmu.2017.00777 (PMC5498468; doi:10.3389/fimmu.2017.00777)

Supplemental information

Exposure to the Epstein-Barr viral antigen LMP1 induces myelin-reactive antibodies *in vivo*

by Lomakin et al.

Supplemental Information includes Supplementary Methods, five Figures and Figure legends

Supplementary Methods

High-pressure nanoLC-MS/MS analysis. Peptide samples were analyzed using a NanoLC Ultra 2D+ nano-HPLC system (Eksigent, USA) coupled via a NanoSpray III ion source (Sciex, Canada) to a TripleTOF 5600+ (Sciex, Canada) mass-spectrometer. The HPLC system was configured in a trap-elute mode. Peptide samples were loaded on a Chrom XP C18 trap column (3 μm 120 Å 350 μm x 0.5 mm; Eksigent, USA) in buffer A (98.9% water, 1% methanol, 0.1% formic acid (v/v)) at a flow rate of 3 μl/min for 10 min and separated on a 3C18-CL-120 HPLC column (3 μm, 120 Å, 75 μm x 150 mm; Eksigent, USA) with a linear gradient of buffer B (99.9% acetonitrile and 0.1% formic acid (v/v)) at a flow rate of 300 nl/min. The gradient was 5-40% of buffer B in 120 min. Between different samples, a blank 45 min run consisting of 5 to 8 min waves (5% B, 95% B, 95% B, 5% B) was required to wash the system and to prevent carryover. Data-dependent MS/MS method included 1 survey MS1 scan followed by 50 dependent MS2 scans. MS1 acquisition parameters were as follows: mass range for MS2 analysis was 300–1250 m/z, signal accumulation time was 250 ms. Ions for MS2 analysis were selected on the basis of intensity with the threshold of 400 cps and the charge state from 2 to 5. MS2 acquisition parameters were as follows: resolution of quadrupole was set to UNIT (0.7 Da), measurement mass range was 200–1800 m/z, signal accumulation time was 50 ms for each parent ion. Collision-activated dissociation was performed with nitrogen gas with collision energy ramping from 25 to 55 V within signal accumulation time of 50 ms. Analyzed parent ions were sent to dynamic exclusion list for 15 sec in order to get an MS2 spectra at the chromatographic peak apex.

Supplementary Figures

Supplementary Figure 1. (A) Western blotting of serum antibodies from MBP-, LMP1- or OVA-immunized mice with bovine MBP, recombinant LMP1 and OVA proteins. (**B**) Schematic representation of the LMP1 molecule and its generated recombinant fragments: transmembrane LMP1-N (3-188 a.a.), cytoplasmic LMP1-C (174-386 a.a.), CTAR1 (188-290 a.a.), CTAR3 (285-330 a.a.) and CTAR2 (325-386 a.a.). (**C**) Analysis of serum IgG levels from MBP- and LMP1-immunized mice specific for LMP1 and its fragments, MBP, MBP81-104 and OVA. Bars represent interquartile range, standard deviation is indicated.

Supplementary Figure 2. Amino acid sequence of unique CDR3 fragments correspond to MBP- and LMP1-reactive IgG from MBP- and LMP1-immunized mice as revealed by *de novo* sequencing of antigen-enriched antibodies.

Supplementary Figure 3. To confirm *de novo* identification of six MS/MS spectra, 11 peptides corresponding to interpretations of these spectra were synthesized. Each figure represents a MS/MS spectrum from the experimental data, and the MS/MS spectrum/spectra of synthetic peptide(s) synthesized to confirm identification of the experimental spectrum. The spectra are marked with red y-series ions, b-series ions are marked with blue. *De novo* identification for 5 out of 6 spectrums was confirmed.
(**A**) Experimental MS/MS spectrum and MS/MS spectra for 2 synthetic peptides: TYGGTFTYDYLNWVK, TYGGTFTDYLYNWVK. Location of the y7-ion on the experimental spectrum suggests correct identification as follows TYGGTFTDYLYNWVK.
(**B**) Experimental MS/MS spectrum and MS/MS spectra for 2 synthetic peptides: GDGSGTSFLLTGGYLK, GDGSGTSFLLTGYGLK. For these two peptides spectra are in

good agreement with the experimental spectrum; it is not possible to choose the most preferential identification.

(**C**) Experimental MS/MS spectrum and MS/MS spectra for 3 synthetic peptides: NTDGSTDYGLLQLGGSR, NTDGSTDYGLLQLGSGR, NTDGSTDYGLLQLSNR. For all 3 peptides spectra are in good agreement with the experimental spectrum, and it is not possible to choose the most preferential identification.

(**D**) Experimental MS/MS spectrum and MS/MS spectra for 2 synthetic peptides: LATSVYASTLTGPVR, LATSVYASTLTGVPR. Lack of y2-ion in the experimental spectrum suggests correct identification as follows LATSVYASTLTGPVR.

(**E**) MS/MS spectrum of the *de novo* identified peptide and MS/MS spectrum of the synthetic peptide ARALETVTLTGK that was proposed as the correct identification.

(**F**) MS/MS spectrum of the peptide identified experimentally, and the MS/MS spectrum of the synthetic peptide: GASDGSTDYGLLQLGGSR. **(G**) Quantitative values of the experimental conformation of the *de novo* identified peptides.

Supplementary Figure 4. Interaction of synthesized biotinylated peptides, representing fragments of determined CDRs, with MBP (**A, B, D, E**) and LMP1 (**C**) monitored by Western blotting analysis (**A**-**C**) and ELISA (**D**, **E**). Data related to peptide origins are shown on panel (**F**). As a negative control, empty blocked agarose or a synthesized biotinylated irrelevant peptide (control peptide) were used.

Supplementary Figure 5. Comparative analysis of relative affinity of anti-MBP and anti-LMP1 antibodies from SJL mice immunized with MBP and LMP1. From each group (n=10) individuals with cross-reactive response were analyzed (n=6 for MBP-immunized mice and n=2 for LMP1-immunizied mice). The relative affinity ratio was calculated as the quotient of ELISA signal representing the level of anti-MBP to anti-LMP1 antibodies in case of MBP-immunized mice, and the level of anti-LMP1 to anti-MBP antibodies for LMP1-immunized mice. Data represent average and standard deviation.

Supplementary figure 1

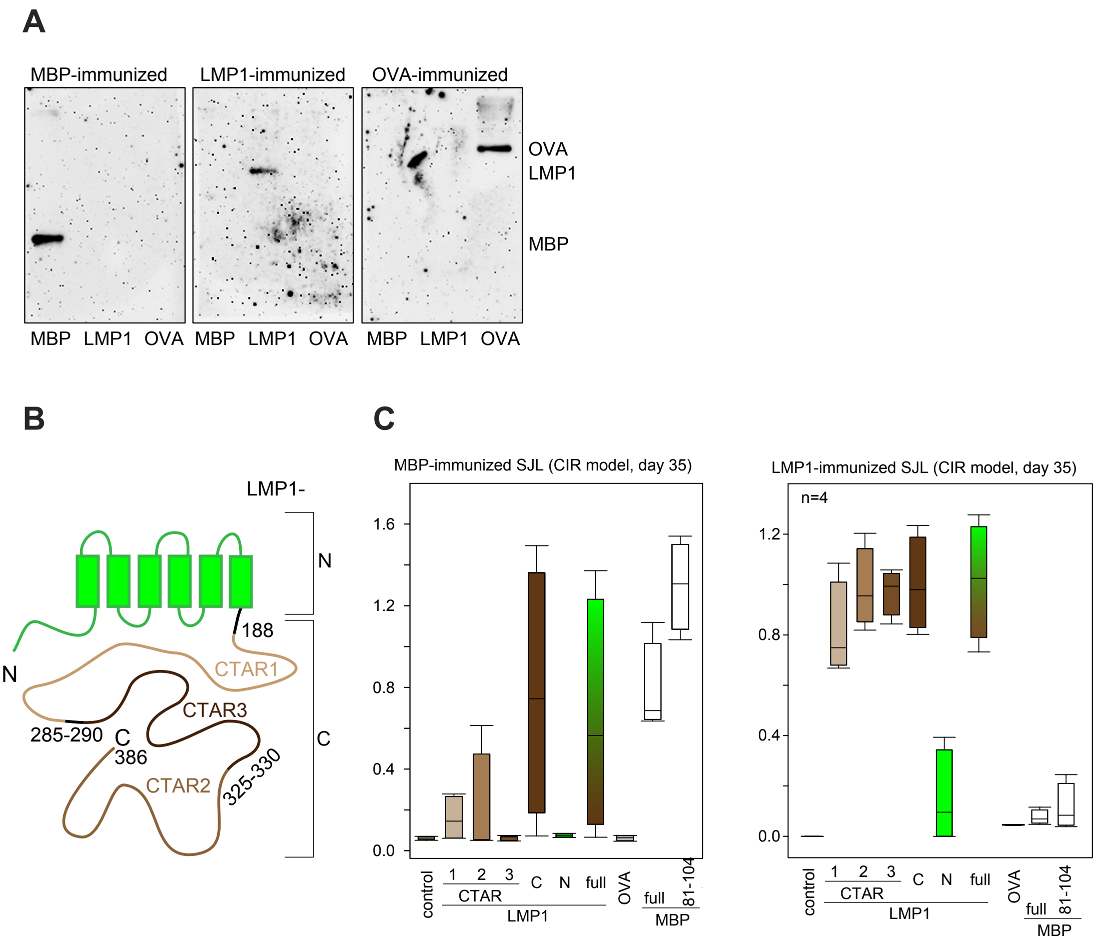

Supplementary figure 2

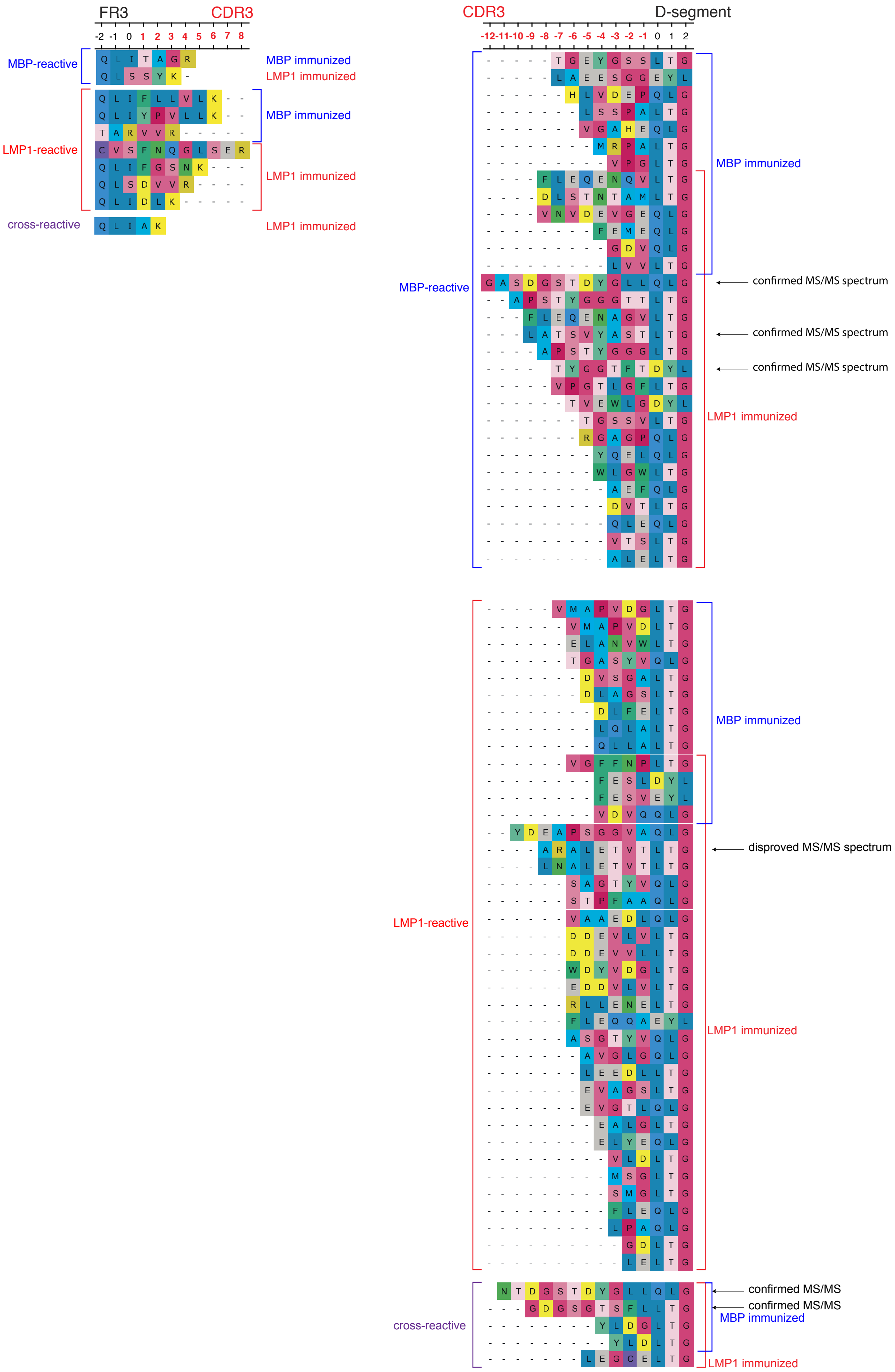

Supplementary figure 3

A

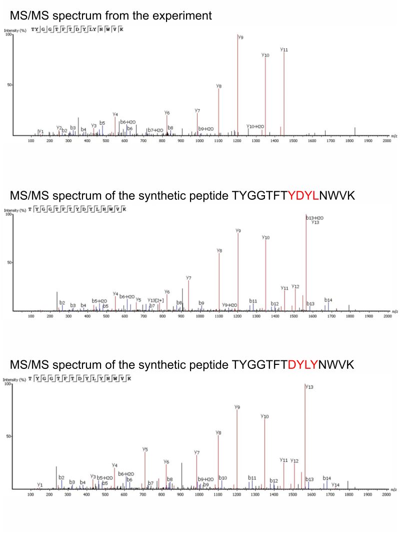

B

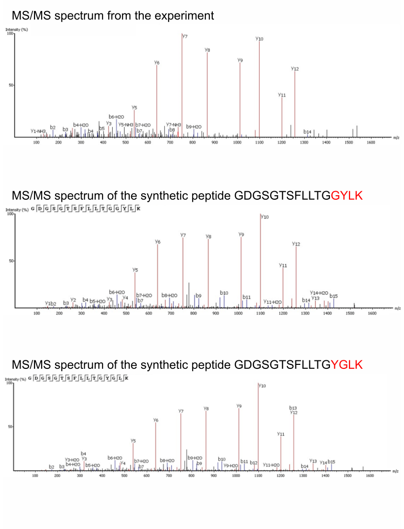

C

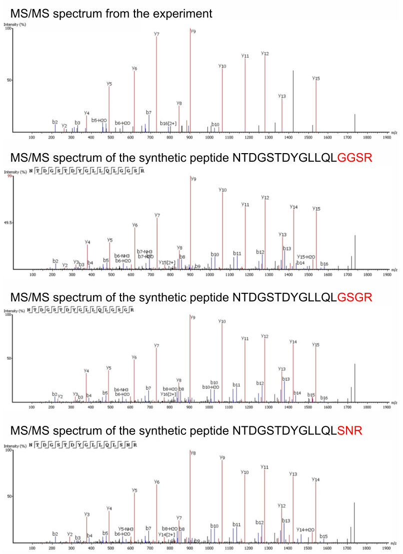

D

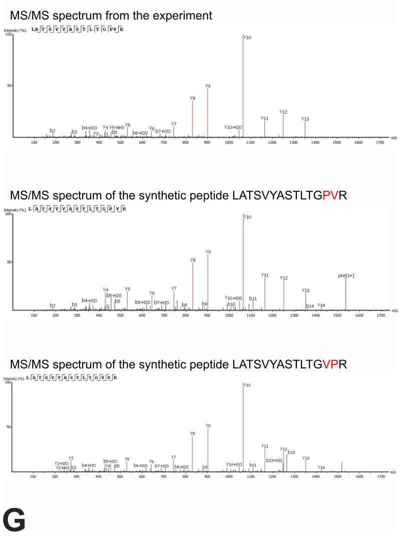

E

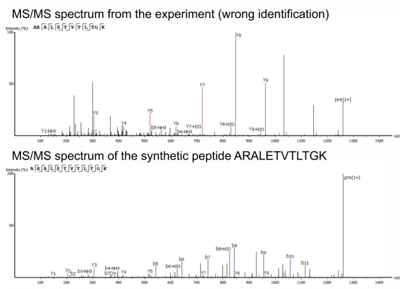

F

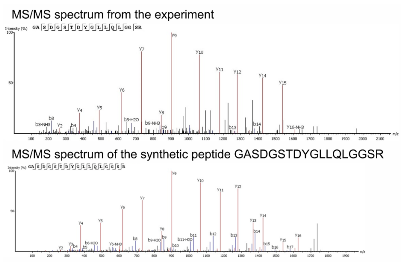

G

Group	Synthesized peptides[1]	Validation scores[2]
A	TYGGTFTYDYLNWVK	0.67
	TYGGTFTDYLYNWVK	0.645
B	GDGSGTSFLLTGGYLK	0.784
	GDGSGTSFLLTGYGLK	0.761
C	NTDGSTDYGLLQLGGSR	0.831
	NTDGSTDYGLLQLGSGR	0.811
	NTDGSTDYGLLQLSNR	0.817
D	LATSVYASTLTGPVR	0.854
	LATSVYASTLTGVPR	0.765
E	ARALETVTLTGK	Identification was not validated
F	GASDGSTDYGLLQLGGSR	0.583

[1]Questionable amino acids, which differs in peptides from one group are colored in red. Fragment of CDR3-segment from identified peptide sequence is underlined
[2]Validation scores > 0.8 – absolutely certain validation; > 0.6 – reliable validation

Supplementary figure 4

A B C

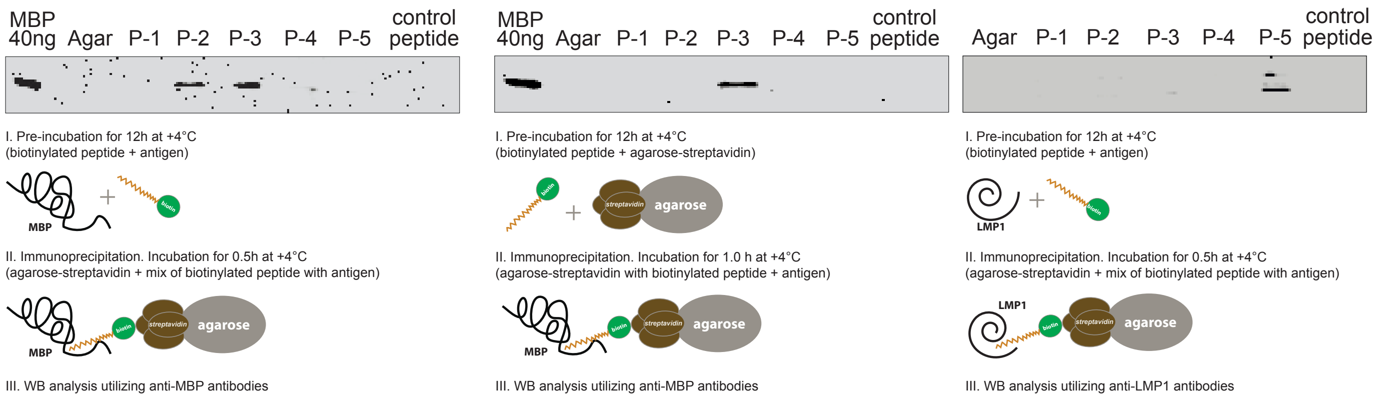

D

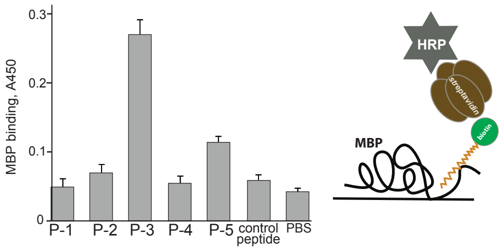

E

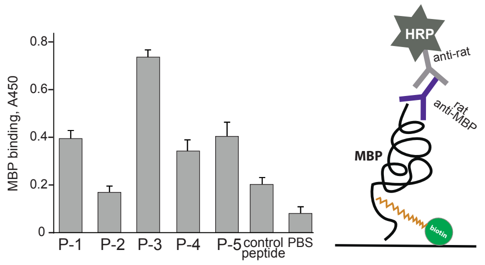

F

ID	Predicted specifity	Origin (antigen of immunization)	Synthesized biotinylated peptides	Experimentally confirmed binding	
				MBP binding	LMP1 binding
P1	MBP-binding	LMP1	GASDGSTDYGLLQLGGSRK(Bio)	-	-
P2		LMP1	LATSVYASTLTGK(Bio)	+/-	-
P3		LMP1	TYGGTFTDYLYNWVK(Bio)	+	-
P4	Cross-reactive	MBP / LMP1	NTDGSTDYGLLQLK(Bio)	-	-
P5		MBP / LMP1	GDGSGTSFLLTGK(Bio)	-	+/-

Supplementary figure 5

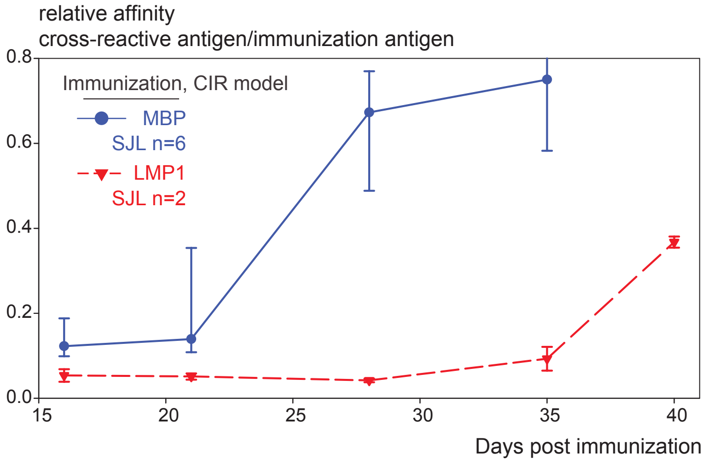

Supplement: Supplementary file 1 [file presentation_1.pdf]
